# Role of hesperetin in LDL-receptor expression in hepatoma HepG2 cells

**DOI:** 10.1186/s12906-016-1165-2

**Published:** 2016-06-27

**Authors:** Nora A. Bawazeer, Hani Choudhry, Mazin A. Zamzami, Wesam H. Abdulaal, Bruce Middleton, Said S. Moselhy

**Affiliations:** Department of Home Economics, Taif University, Taif, Saudi Arabia; Department of Biochemistry, Faculty of Science, Cancer and mutagensis unit, Center of Innovation in Personalized Medicine, King Fahd Center for medical Research, King Abdulaziz University, Jeddah, Saudi Arabia; Department of Biochemistry, Cancer and mutagensis Unit, King Fahad Medical Research Center, KingAbdulaziz University, Jeddah, Saudi Arabia; Department of Biochemistry, Medical School, Nottingham University, Nottingham, UK; Department of Biochemistry, Faculty of Science, King Abdulaziz University, Jeddah, Saudi Arabia; Department of Biochemistry, Faculty of Science, Ain shams University, Cairo, Egypt

**Keywords:** LDL-receptor, Hesperetin, HepG2 cell line

## Abstract

**Background:**

High plasma concentration of low-density lipoprotein cholesterol (LDL-c) plays a significant role in the incidence of atherosclerosis and coronary heart diseases. The aim of this study was to investigate the mechanism by which the citrus flavonoid, hesperetin, regulates the LDL receptor (LDLr) gene in the human liver using the human hepatoma cell line, HepG2.

**Methods:**

Luciferase reporter gene assays were performed (in the absence of lipoprotein) to measure the activity of the LDLr promoter and the promoters of the sterol regulatory element binding protein (SREBP) transcription factors that control the LDLr promoter.

**Results:**

Only SREBP-1 promoter activity was significantly increased 4 h after exposure to 200 μM hesperetin. However, after 24 h incubation with 200 μM hesperetin, the activities of all the promoter-constructs, SREBP-1a, -1c, -2 and LDLr, were significantly increased. The effects of 200 μM hesperetin on elevating LDLr mRNA levels were possibly due to regulation of LDLr gene transcription by SREBP-la and SREBP-2.

**Conclusions:**

We conclude that 200 μM hesperetin was likely to have stimulated LDLr gene expression in human hepatoma HepG2 cells via increased phosphorylation of PI3K andERK1/2, which increased SREBP-1a and SREBP-2 mRNA levels and enhanced the maturation of the encoded proteins. This may lead to lower plasma LDL cholesterol; therefore, diets supplemented with hesperidin might provide cardio-protective effects and reduce mortality and morbidity from coronary heart diseases.

## Background

High levels of plasma LDL-c are associated with accelerated atherosclerosis [1]. There is strong evidence supporting the involvement of oxidized LDL in cholesterol loading of macrophages, foam cell formation and atherogenesis [2]. The antioxidant properties of flavonoids result from the formation of stable radicals, due to electronic delocalization through the hydroxyaromatic groups in the A, B and C rings. It has been reported that biological activity of flavonoids, which are directly related to electronic delocalization, may be affected by the presence/absence of the C2–C3 double bond, the position of hydroxyl groups or the planarity of the molecular structure [3].

Oxidation of LDL by macrophage free radicals leads to depletion of endogenous α- tocopherol levels causing the level of lipid hydroperoxides to rise. As a result, macrophages uptake of oxidized LDL is increased. Although it is believed that flavonoids can inhibit LDL oxidation by macrophages, the mechanism is unknown. However, several possibilities may explain flavonoid function. One possibility is that flavonoids protect α-tocopherol from oxidized LDL from being consumed. This may occur either by scavenging free radicals or by converting α-tocopheroxyl radicals into α-tocopherol. Another possibility is that flavonoids may diminish the formation or release of free radicals by cells. In addition, flavonoids may inhibit LDL oxidation by scavenging superoxide anions, hydroxyl radicals or lipid peroxyl radicals. It has also been found that flavonoids have the ability to bind iron and copper [4].

Cholesterol feeding in animals can reduce the specific binding of LDL to hepatic membranes and experiments with cultured rat hepatocytes have suggested that this regulation is mediated by the uptake of cholesterol from exogenous lipoprotein via the LDLr, as is the case in cultured human skin fibroblasts. However, human hepatoma cells express LDL receptor activity in the presence of lipoproteins, suggesting that the suppression of LDLractivity in hepatocytes is less responsive to the external concentration of lipoprotein cholesterol than in fibroblasts [5].

Hesperetin belong to a class of flavonoids called flavanones. Flavanones occur almost exclusively in citrus fruits. Although the highest concentrations are found in the solid tissues, several hundred milligrams per liter are present in the juice. Hesperidin (hesperetin-7-rutinoside) is the major flavonoid of oranges, found largely as glycosides, which are hydrolyzed to their active forms by intestinal bacteria [6]. Sweet orange juice contains narirutin (30–84 mg/L) and hesperidin (235–407 mg/L) [7–9]. SREBPs are members of the basic helix-loop- helix-leucine zipper (bHLH-Zip) family of transcription factors thatdirectly activate the transcription of more than 30 genes dedicated to the biosynthesis and uptake of cholesterol, fatty acid, triglycerides and phospholipids.

However, in the liver, SREBPs also regulate plasma lipoproteins and bile micelle synthesis genes [10]. Several distinct genes of both cholesterol and fatty acid metabolism aredirectly activated by SREBPs in cultured cells [11]. In vivo, genes of cholesterol metabolism are activated by SREBP-2, while genes of fatty acid and triglyceride metabolism are activated by SREBP-1c [12].

Although plant flavonoids have many potent biological properties, for example to lower blood cholesterol and as anticancer, antiviral, antioxidant drugs, their mechanisms of action have not been fully elucidated. The aim of this study was to investigate the mechanism(s) by which hesperetin may regulate the activity of the LDLr promoter, in human liver using human hepatoma HepG2 cells and to investigate whether these compounds act via a SREBP-dependent mechanism or as a result of modulation of other signal transduction pathways.

## Methods

### Cell culture and transfection

HepG2 human hepatoma cells were obtained from the American type Culture Collection (ATCC). Cells were maintained in 10 % Growth Medium (GM), consisting of Dulbecco’s Modified Eagle’s Medium (high glucose with 4500 mg/L glucoseand sodium bicarbonate without L-glutamine; DMEM; Sigma) supplemented with 10 % (v/v) fetal Bovine serum (FBS; Sigma), antibiotics (penicillin 100U/ml, 100 μg/mlstreptomycin sulphate) and 2 mM L-glutamine (Sigma). Adherent HepG2 and McARH-7777 cells were maintained in monolayer culture in 75 cm^2^ flasks in 10 % GM and incubated at 37 °C, in 5 % CO_2_ for HepG2 and 10 % CO_2_for McARH-7777 cells. Fresh GM was added every 2 days and cells were sub-cultured once a week by trypsinisation when the cells were 70 %–80 % confluent. The medium was removed by aspiration and the cells washed with 3 ml 1x EDTA/saline. One milliliter of 1x trypsin solution was added and the flask incubated at 37 °C for 2–3 min. Ten milliliters of 10 % GM was added and the cells were harvested by centrifugation at 900 × gfor 5 min. The cell pellet was resuspended in fresh 10 % GM. Cells were split 1:2 to 1:7 into 75cm^2^ flasks for growth or into 6-well plates (9.6 cm^2^/well; IWAKI) for experiments. Hesperetin (purity: >98 %, was purchased from Sigma).

### Transfection for dual-luciferase assay

Cells were transiently transfected with 1 μg/well human pLDLr Luc^+^, pSREBP-1a Luc+, pSREBP-1c Luc + and pSREBP-2 Luc^+^ using GeneJuice transfection reagent (Novagen) according to the manufacturer's protocol. Cells were then incubated in the transfection medium for 4 h. Medium was then removed and cells were washed with 2 ml/well basal DMEM. Cells were then exposed to DMSO as a vehicle or hesperetinin fresh 5 mg/ml Human lipoprotein-deficient serum (LPDS). After 24 h incubation, cells were lysed for Firefly Luciferase and protein assays.

### Dual-luciferase reporter assay system

Aluminometer (TD-20/20) was programmed to perform a 2 s pre-measurement delay, followed by a 10s measurement period for each reporter assay. One hundred microliters of LAR II was pre-dispensed into a luminometer tube and then 20 μl of cell lysate was added. After mixing by pipetting two or three times, the tube was placed in the luminometer and the firefly luciferase activity measurement was recorded. Then, 100 μl of Stop & Glo Reagent was added and vortex-mixed briefly. The Renilla luciferase activity was measured.

### BioRad protein assay

Cell lysates were diluted (1:20) in 1x PLB. Tenmicrolitersfrom each sample was assayed for protein concentration using Bovine Serum Albumin (BSA, Sigma) protein standards of known protein concentration (0, 1, 2, 3, 4 and 5 mg/ml). Bio-Rad Reagent was diluted 1:5 (v/v) with distilled water, 200 μl was added to each sample and to the standards in a 96-well plate (Corning Incorporated, Costar). The plate was then carefully mixed to remove bubbles and incubated for 5 min at room temperature and the absorbance was read using a Dynatech MR 5000 Microplate Reader at a wavelength of 595 nm. Protein concentrations (mg/ml) were calculated from the standard curve and used to normalize the luciferase activity values [13].

### DNA quantitation

A Gene Quant2 spectrophotometer (Pharmacia) or a NanoDrop ND-1000 Spectrophotometer (NanoDrop Technologies) was used to estimate the concentration of the double strand DNA by measuring absorbance at 260 nm and 280 nm. The ratio of absorbencies was used to assess the purity of DNA. The absorbance at 260 nm was used to calculate DNA concentration using the following equation: Concentration (μg/ml) = dilution factor x A_260_× 50 μg/ml. A solution with O.D_260_ = 1.0 contains approximately 50 μg/ml of double stranded DNA. Pure DNA preparations have a ratio at 260/280 of 1.8–2.0 (Qiagen, 2006).

### Polymerase chain reaction (PCR)

A standard PCR protocol was used to amplify specific sequences of DNA for molecularanalysis.

### Statistical analysis

The computer software program GraphPad Prism (GraphPad 4.01) was used for all data analysis. A one-way analysis of variance (ANOVA) was used to compare significant differences between groups. *Post hoc* Dunnett’s or Bonferroni’s Multiple Comparison tests were used. The Statistical significance was accepted if the null hypothesis was rejected with a *p* < 0.05. Results are expressed as the mean ± SEM of separate experiments. All analyses were carried out with 95 % confidence intervals.

## Results

**Fig. 1:**
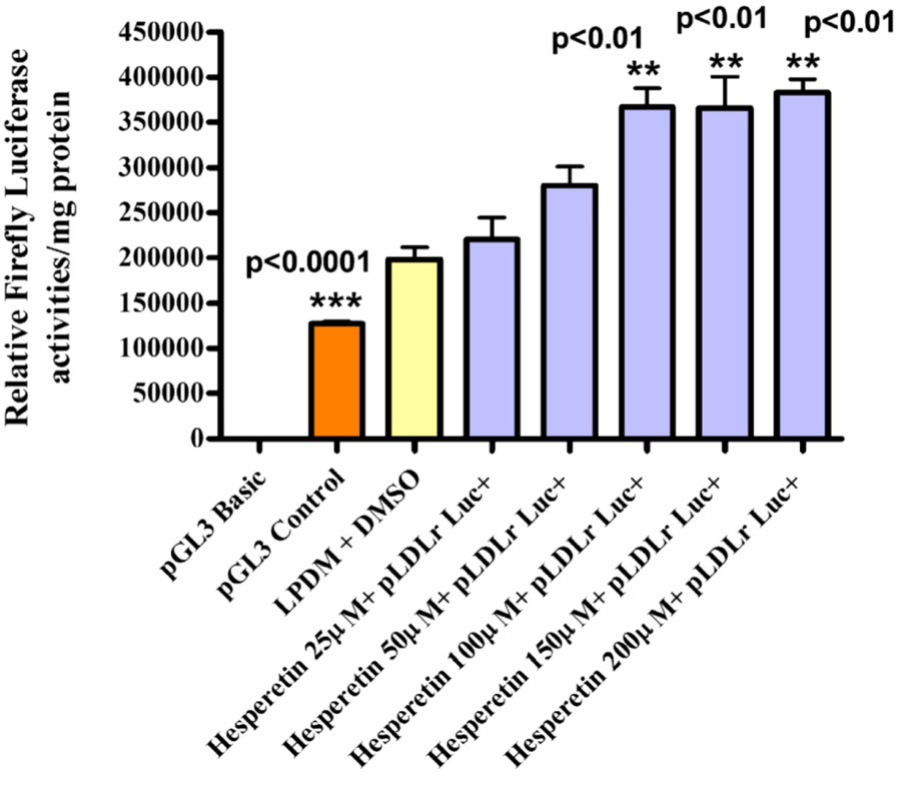
The Effect of Different Hesperetin Concentrations in LPDM on the Activity of pLDLr Luc + in Human Hepatoma HepG2 Cells after 24 h. HepG2 Cells were transfected with 1 μg/well human pLDLr driving firefly luciferase. To assay the pLDLr promoter response to the different concentrations of hesperetin, 5 mg/ml LPDM + 2 μl/ml DMSO was used as vehicle. Cells were exposed to 25 μM, 50 μM, 100 μM, 150 μM and 200 μM hesperetin in fresh 5 mg/ml LPDM. After incubation for 24 h, cells were harvested for luciferase and protein assays. pGL3 Luciferase reporter-Basic and -Control vectors were used to indicate the transfection efficiency. The difference between groups was evaluated by one-way ANOVA with Dunnett’s multiple comparisons test. The data represent an individual experiment, which was repeated twice. Values were expressed as mean ± SEM; *n* = 3 per treatment

Results presented in Fig. 1 show the effect of different hesperetin concentrations in lipoprotein deficient medium (LPDM) on the activity of pLDLr Luc + in human hepatoma HepG2 cells after 24 h. HepG2 Cells were transfected with 1 μg/well human pLDLr, which drives Firefly Luciferase. To assaythe pLDLr promoter response to different concentrations of hesperetin, 5 mg/ml LPDM + 2 μl/ml DMSO was used as vehicle. Cells were exposed to 25 μM, 50 μM, 100 μM, 150 μM and 200 μM hesperetin in fresh 5 mg/ml LPDM. After incubation for 24 h, cells were harvested for Luciferase and protein assays. pGL3 Luciferase reporter-Basic and -Control vectorswere used to indicate the transfection efficiency. The difference between groups was evaluated by one-way ANOVA with Dunnett’s Multiple Comparisons Test. The data represent an individual experiment, which was repeated twice. Values areexpressed as mean ± SEM; n = 3 per treatment. Hesperetin at 25 μM and 50 μM increased pLDLr Luc + activity compared with the vehicle but the increase was not significant, while 100 μM, 150 μM and 200 μM hesperetin significantly up-regulated pLDLr Luc + activity after 24 h (** *p* < 0.01).

HepG2 cells were transiently transfected with the human pSREBP-1a promoter driving firefly luciferase. After transfection, spent medium was removed and cells were washed with 2 ml/well basal DMEM. Cells were then exposed to DMSO vehicle or to 25 μM, 50 μM, 100 μM, 150 μM and 200 μM hesperetin in 5 mg/ml fresh LPDM. After incubation for 24 h, the cells were lysed for luciferase and protein assays. Hesperetin at 25 μM and 50 μM caused a slight increase in the pSREBP-1a Luc + activitycompared with the vehicle. However, hesperetin concentrations of 100 μM (* *p* < 0.05), 150 μM (* *p* < 0.05) and 200 μM (** *p* < 0.01) significantly up-regulated pSREBP-1a Luc + activity in HepG2 cells.

**Fig. 2:**
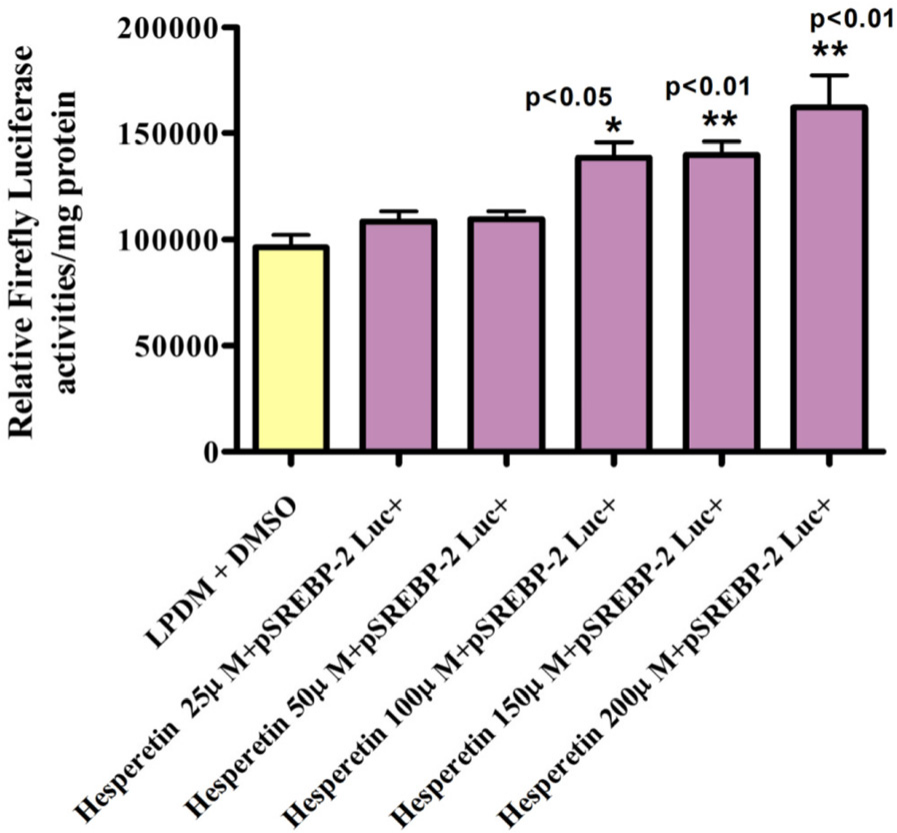
The Effect of Different hesperetin Concentrations in LPDM on the Activity of pSREBP-2 Luc + in Human Hepatoma HepG2 Cells after 24 h. HepG2 Cells were transfected with 1 μg/well human pSREBP-2 driving firefly luciferase. 5 mg/ml LPDM + 2 μl/ml DMSO was used as vehicle. Cells were exposed to 25 μM, 50 μM, 100 μM, 150 μM and 200 μM hesperetin in fresh 5 mg/ml LPDM. After incubation for 24 h, cells were harvested for luciferase and protein assays. The difference between groups was evaluated by one-way ANOVA with Dunnett’s multiple comparisons test. The data represent an individual experiment, which was repeated twice. Values were expressed as mean ± SEM; *n* = 3 per treatment

Figure 2 shows the effect of different hesperetin concentrations on the activity of pSREBP-2 Luc + in HepG2 cells 24 h after transfection with 1 μg/well human pSREBP-2 driving firefly luciferase.2 μl/ml DMSO was used as vehicle. Cells were exposed to 25 μM, 50 μM, 100 μM, 150 μM and 200 μM hesperetin. After incubation for 24 h, cells were harvested for luciferase and protein assays.

**Fig. 3:**
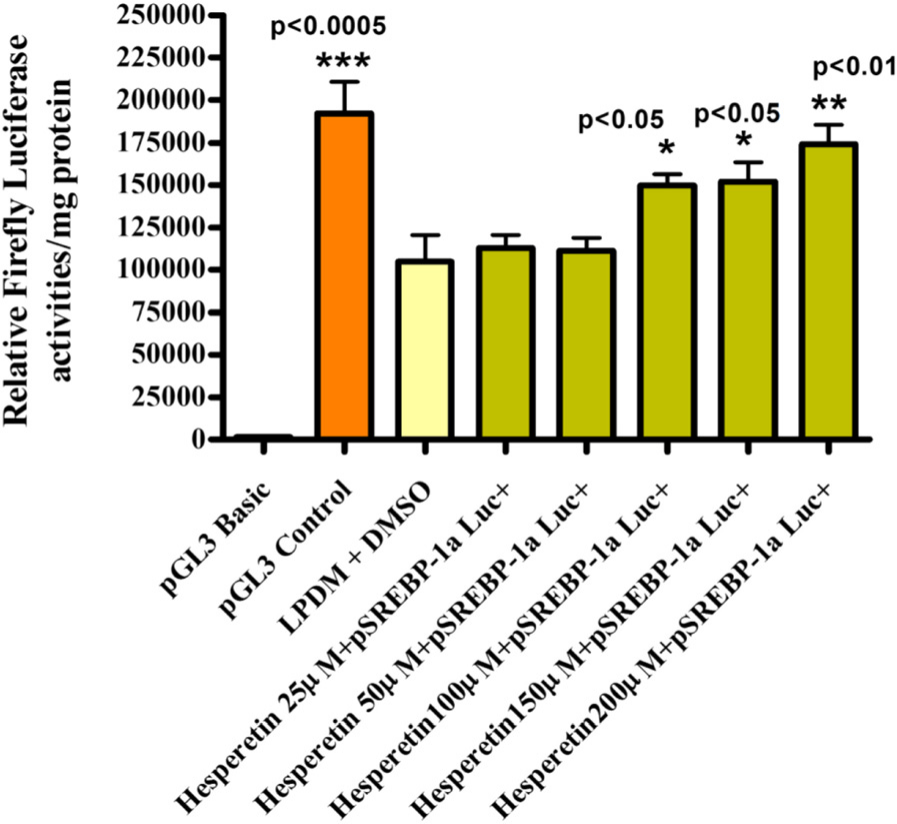
Effect of Different hesperetin Concentrations in LPDM on the Activity of pSREBP-1c Luc + in Human Hepatoma HepG2 Cells after 24 h. HepG2 Cells were transfected with 1 μg/well human pSREBP-1c driving firefly luciferase activity. Cells were exposed to 50 μM,100 μM,150 μM and 200 μM hesperetin in fresh 5 mg/ml LPDM. After incubation for 24 h, cells were harvested for luciferase and protein assays. The difference between groups was evaluated by one-way ANOVA with Dunnett’s multiple comparisons test

**Fig. 4:**
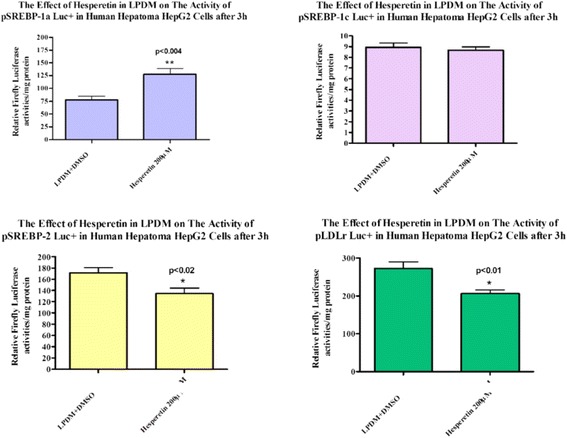
Effect of hesperetin in LPDM on the Activity of Human pSREBP-1a Luc+, pSREBP-1c Luc+, pSREBP-2 Luc + and pLDLr Luc + in Human Hepatoma HepG2 Cells after 3 h. HepG2 cells were transiently transfected in LPDM with 1 μg/well human pLDLr Luc+,pSREBP-1a Luc+, pSREBP-1c Luc + or pSREBP-2 Luc + as described above. Firefly luminescence was normalized to total cellular protein to determine promoteractivity

The results (Fig. 3) also indicated that 200 μM hesperetin significantly decreased the activity of the MTP promoter in HepG2 cells. Hesperetin at 200 μM significantly up-regulated the mRNA levels of both SREBP- 1a and LDLr mRNAs after 4, 8, 12 and 24 h, while the levelsof SREBP-2 mRNA were significantly increased after 12 and 24 h. However, the mRNA level of SREBP-1c was significantly down-regulated after 4, 8, 12 and 24 h, while fatty acid synthase mRNA expression was significantly decreased after 8, 12 and 24 h. Both HMG-CoA reductase and acetyl-CoA carboxylase-α mRNA levels were also significantly decreased after 12 and 24 h (Fig. 4).

## Discussion

The regulation of hepatic LDL-c catabolism relies on the activity of the LDL receptor (LDLr) to maintain a steady-state plasma concentration of LDL-c [14]. In the human body, the liver is the most LDL-receptor abundant organ and accounts for 80 %–90 % of the total LDL clearance from plasma [15]. The identification of the sterol regulatory element binding protein (SREBP) transcription factors furthered understanding of cellular cholesterol homeostasis [16]. In vivo, SREBPs are found to be crucial for the synthesis and clearance of atherogenic lipoproteins and also for the highly activated transcription of several genes, including the LDLr [17].

SREBPs are members of the basic helix-loop-helix-leucine zipper (bHLH-Zip) family of transcription factors that directly activate the transcription of more than 30 genes dedicated to the biosynthesis and uptake of cholesterol, fatty acid, triglycerides and phospholipids [18].

SREBP-1a, SREBP-1c and SREBP-2 are the three major SREBP isoforms to have been identified. They are synthesized in the liver as precursors membrane-bound to the endoplasmic reticulum and nuclear envelope [19]. Low intracellular cholesterol concentration promotes atwo-step proteolytic cleavage process for SREBP-l and SREBP-2 precursors. As a result, transcriptionally active SREBP migrates to the nucleus where it strongly activates transcription of several genes involved in cholesterol and fatty acid synthesis [20]. LDLr uptake of cholesterol increases intracellular cholesterol, which inhibits the release of mature SREBP resulting in suppression of LDLr transcription [21]. Deletion of the acidic NH_2_-terminal region of SREBP, which is the transcriptional activation domain, prevented the transcriptional activation of the LDLr. High-level expression of LDLr is achieved when SREBP-l and SREBP-2 bind to the sterol response element-1 (SRE-1) sequence and interact with repeat 3 of stimulating protein-1 (Sp1), thereby reducing elevated levels of plasma cholesterol [22]

In vivo studies revealed that SREBP-2 activates cholesterol metabolismgenes, whereas SREBP1c activated genes of fatty acid and triglyceride metabolism. However, SREBP-1a seemed to activate both pathways. Consumption of fruits and vegetables can protect against the development of cardiovascular diseases [23]. Although plant flavonoids can act as anticancer, antiviral and antioxidant agents and can lower blood cholesterol, the mechanisms of these actions in most cases have not been fully elucidated [24].

As the liver plays a major role in cholesterol and lipid metabolism, human hepatoma HepG2 cells, a human liver-derived cell line [25], was used as a model to investigate the molecular mechanisms by which the citrus flavonoid, hesperetin, regulates hepatic activity of genes associated with cholesterol and lipid metabolism.

LDL cholesterol reduction occurs mainly via LDLr up-regulation. LDLr expression is mainly regulated at the transcriptional levelvia SREBP-1a, -1c, and -2 [26]; therefore, to investigate the molecular mechanism by which hesperetin regulates LDLr transcription luciferase-reporter gene assays were performed to compare the transcriptional regulation of the SREBP-1a, -1c, and -2 promoters on the activity of the LDLr gene. Human lipoprotein-deficient serum (LPDS) was used to maximize LDLr activity.

Preliminary experiments were performed to examine LDLr promoter sensitivity to the addition of cholesterol or LPDM. Transient transfection data revealed that LDLr promoter activity was significantly elevated in cells incubated in LPDM as compared with complete growth medium. This was in agreement with several studies which revealed that LDLr transcription activity was activated in the absence of sterols [27]. The effect of hesperetin in LPDM on regulating the activity of LDLr, SREBP-1a, SREBP-1c and SREBP-2 was dose-dependent with notable stimulation at 100 μM and 200 μM after 24 h. Hesperetin at 200 μM appeared to be potent in up-regulating all promoters investigated. Cell viability assays confirmed that 200 μM hesperetin did not cause any harmful effect in HepG2 cells; therefore we used it in all our experiments.

Time-course transient transfection data indicated that early significant activation of SREBP-1a promoter was observed after 3 h incubation with 200 μM hesperetin. However, the gene expression activities of LDLr, SREBP-1a, SREBP-1c and SREBP-2 were increased significantly after incubation for 24 h with 200 μM hesperetin. Based on our findings we suggest that only SREBP-1a and SREBP-2 are involved in the regulation of LDLr expression in HepG2 cells as previously reported by many studies [28]. It would also appear that the SREBP-1c promoter is less likely to be involved in the regulation of LDLr expression in HepG2 cells because in previous studies its electively stimulated fatty acid synthesis and insulin-induced glucose metabolism [29]. The extremely low mean luciferase activity of the SREBP-1c promoter construct compared with the other constructs could be explained by reduced SREBP-1c mRNA expression and an undetectable level of SREBP-1c protein. These findings determined that 200 μM hesperetin did not increase SREBP-1c promoter activity in HepG2 cells. Hesperetin at 200 μM also significantly decreased the activity of the MTP promoter in Human Hepatoma HepG2 cells.

The investigation of mRNA levels by qRT-PCR (Taqman) assay in HepG2 cells revealed that 200 μM hesperetin in LPDM significantly up-regulated the mRNA levels of both the SREBP- 1a and LDLr mRNAs after 4, 8, 12 and 24 h, while the expression of SREBP-2 mRNA was significantly increased after 12 and 24 h. However, the mRNA level of SREBP-1c was significantly down-regulated after 4, 8, 12 and 24 h, while the fatty acid synthase mRNA expression was significantly decreased after 8 12 and 24 h. Both the HMG-CoA reductase and Acetyl-CoA Carboxylase-α mRNA expressions were also significantly decreased after 12 and 24 h. Our results suggest that it is likely that the effects of 200 μM hesperetin on elevating LDLr mRNA are due to regulation of gene transcription by SREBP-la and SREBP-2 in HepG2 cells because in the absence of sterols, high-level expression of the LDLr was achieved when SREBP-l and SREBP-2 were bound to the SRE-1 sequence and interacted with Sp1 [30–32].

In this study, it is likely that the suppression effect of hesperetin on hepatic SREBP-1c mRNA levelsis associated with the reduction in mRNA levelsof both acetyl-CoA carboxylase and fatty acid synthase. Furthermore, 200 μM hesperetin in LPDM had no effect on the SREBP-1a precursor form. However, they both caused a significant increase in the amount of SREBP-1a mature form which could be a result of phosphorylation by PI3K and ERK1/2. Thus, the effects of hesperetin upon the expression of the LDLr gene are likely to occur via an increased SREBP1a and SREBP-2 mRNA levels and increased maturation of the encoded proteins.

## Conclusion

We conclude that, 200 μM hesperetin is likely to stimulate LDLr gene expression via increase phosphorylation of PI3K and ERK1/2, which enhance mRNA levels of the transcription factors, SREBP-1a and SREBP-2, and increased their protein maturation in human hepatoma HepG2 cells. Since this may lead to lower plasma LDL cholesterol, diets supplemented with hesperetin may produce cardio-protective effects in humans and reduce mortality and morbidity from coronary heart diseases.

## Abbreviations

DMEM, Dulbecco’s Modified Eagle’s Medium; GM, growth medium; HepG2, human hepatoma cell line; LDL-c, LDL cholesterol; LDLr, LDL receptor; LPDM, lipoprotein deficient medium; MAPK, mitogen-activated protein kinase; PI3K, phosphatidylinositol 3-kinase; SRE, sterol response element; SREBP, sterol regulatory element binding protein
